# Social behavior is perturbed in mice after exposure to bisphenol A: a novel assessment employing an IntelliCage

**DOI:** 10.1002/brb3.130

**Published:** 2013-02-20

**Authors:** Hiroshi Ogi, Kyoko Itoh, Shinji Fushiki

**Affiliations:** Department of Pathology and Applied Neurobiology, Graduate School of Medical Science, Kyoto Prefectural University of MedicineKyoto, Japan

**Keywords:** Bisphenol A, development, IntelliCage, mice, social behavior

## Abstract

In order to investigate whether or not prenatal and lactational exposure to bisphenol A (BPA) affects social behavior in mice, pregnant mice were exposed to 500 μg/kg of BPA daily from embryonic day 0 (E0) until postnatal day 21 (P21). The behavior of offspring was monitored at 11–13 and 13–15 weeks of age using an automated behavior assessment system (IntelliCage). Groups of eight mice were tasked with a nose poke, which enabled the mice to open a door to drink bottled water at the corner of their cage. BPA-exposed females visited the corner without drinking behavior during the light cycle less frequently than control female mice did. BPA-exposed males stayed at the corner for longer periods of time and showed a significantly stronger bias in the visit with drinking. In addition, the BPA-exposed males showed a shorter time interval before they visited the corner after preceding animals had visited it, compared with the control males. These findings suggest that prenatal and lactational BPA exposure might affect murine motivational behavior in a social setting differently in males and females.

## Introduction

Bisphenol A (BPA) is an endocrine-disrupting chemical, widely used in manufacturing plastic products and epoxy resins. Humans are exposed ubiquitously to this chemical. In addition to effects on the reproductive system, there is growing concern that intrauterine exposure affects brain development, behavior, and emotions. The results of several studies have suggested that fetal and/or lactational exposure to BPA alters the behavior of offspring in rodents (Gioiosa et al. 2007; Yu et al. 2011; Nakamura et al. 2012). Other studies suggested that changes in neurotransmitters might underlie those behavioral changes (Negishi et al. 2004; Ishido et al. 2007; Tando et al. 2007; Nakamura et al. 2010). Among the various effects of BPA on behavior, the social and emotional domains have been especially noticeable. In humans, one study showed a positive association between a high maternal urinary concentration of BPA during gestation and behavior problems, including anxiety- and depression-based behavior in 3-year-old girls (Braun et al. 2011). It has also been reported that there are differences in the effects of exposure to BPA between boys and girls (Braun et al. 2011; Perera et al. 2012).

Recently, the IntelliCage (a fully automated behavioral phenotyping device) has been utilized in the evaluation of the behavior of laboratory animals in order to eliminate human interference (Krackow et al. 2010; Endo et al. 2011). In addition to eliminating human interference, the use of an IntelliCage can be advantageous in the assessment of long-term spontaneous behavior of group-housed animals.

In this study, we attempted to address the questions of how prenatal and neonatal exposure to BPA affects nonsexual behavior, including social behavior and preference formation. In order to achieve our study goals, we orally administered BPA to dams during pregnancy and lactation, and thereafter we evaluated various indices of group-housed offspring with an IntelliCage.

## Materials and Methods

### Animals and treatments

C57BL/6J mice (CLEA Japan, Tokyo, Japan) were housed in a controlled temperature (24°C), lighting (12-h light/dark cycle), and humidity (40–60% RH) environment with free access to food and water. All the animal studies were approved by the Institutional Review Board for Biomedical Research using Laboratory Animals at Kyoto Prefectural University of Medicine, and the animals were handled in accordance with the institutional guidelines and regulations.

Adult females were mated and the morning when a vaginal plug was observed was designated embryonic day 0 (E0). The dams were dosed daily by feeding tube with 500 μg/kg body weight/day of BPA (Wako, Osaka, Japan) dissolved in 0.01% ethanol for the BPA-exposure group (BPA group) or the same amount of 0.01% ethanol for the vehicle control group (control group) from E0 to 3 weeks after delivery. The dosage 500 μg/kg body weight/day of BPA is 100 times less than the no observed-adverse-effect level (NOAEL; 50 mg/kg/day).

The offspring were weaned at postnatal week three (P3W) and housed separately for each sex (2–5 mice in each cage) until P11W for the females or P13W for the males. All animals were fed standard rodent diet CE-2 (CLEA Japan, Tokyo, Japan) upon arrival and for the duration of the experiment. We prepared three separate animal groups, two control groups and one BPA-exposure group. In the first control cohorts, eight female and eight male pups were randomly chosen from three dams avoiding pups of extremely low or high body weight. In the second control cohorts, eight female pups were randomly chosen from five dams and eight male pups were chosen from four dams. BPA cohorts had six dams. Eight female pups were randomly chosen from four dams and eight male pups were chosen from five dams.

### Behavioral assessment with the IntelliCage

#### Apparatus

The IntelliCage (NewBehavior AG, Zurich, Switzerland) is a novel system for automated monitoring of the spontaneous cognitive and learning behavior of mice living in social groups.

The system fits into a large standard laboratory rodent cage (20.5 cm high, 62 × 44 cm at the top and 55 × 38.5 cm at the base). The system provides four recording chambers that fit into the corners of the housing cage, covering a right-angle triangular 15 × 15 × 21 cm floor space. Only one mouse at a time can access a single chamber via a plastic tube in which an antenna code-reading transponder is embedded. The 13-mm-diameter openings are placed on the left and right side of the corners and each gives access to the nipple of one water bottle. These openings are equipped with light-beam nose poke sensors that detect nose pokes by interruption of the light beam. The IntelliCage is controlled by a single PC. Several programs allow the experimenter to create the files for the conditioning schedules, to run them, and analyze the data obtained.

#### Experimental protocols

A behavioral experiment was performed sequentially for each cohort in one cage. First, the control female cohort was tested, followed by the control male cohort. After that, a similar experiment was conducted for the BPA group, with the mice at the same age as in the control group. Finally, the second control group was assessed.

Five days prior to the initiation of the IntelliCage experiment, eight pups were chosen for assessment and subcutaneously implanted with a glass-covered transponder with unique ID codes for radio-frequency identification–based animal identification (Datamars SA, Bedano, Switzerland) under isoflurane anesthesia. The assigned animals were housed in two cages.

The IntelliCage experiment was started at 1900h, composed of two sessions:

1. Animals were introduced to the IntelliCage and were allowed free access to all water sources for 3 days.
2. “NP1” On the fourth day, all water-access doors were initially closed, so that mice had to perform nose pokes, which enabled them to open a respective door (doors were closed automatically 7 sec after a nose poke) for drinking.

The NP1 session lasted 12.5 days, ended at 0700h.

#### Statistical analysis

We ran statistical analysis with JMP 10.0.0 (SAS Institute Inc., Cary, NC). We performed Tukey's HSD test between the control group (16 pooled values) and the BPA group (eight values) separately for each sex. Wilcoxon rank sum tests were employed to analyze differences for sex and other indices and parameters. The statistical analysis procedures did not exclude extreme values.

## Results

### Visit number and duration

In order to evaluate behavioral differences between the groups, we extracted the visit number and the duration throughout the entire period of the experiments as basic indices. Indices were evaluated with two parameters, including (1) the evaluated period as nocturnal and diurnal, and (2) the total visit with/without drinking. Because the drinking duration depended on the amount of water drunk by each animal, we subtracted the drinking duration from the visit duration in order to focus on nonphysiological phenomenon.

Table 1 showed the 1 day average number of visits for all sessions. In all the groups, the animals visited the corners more during nocturnal period than during diurnal period (control females total and control males total: *P* < 0.0001, BPA females total and BPA males total: *P* < 0.001, Wilcoxon rank sum test), and the number of visits without drinking was significantly higher than those of visits with drinking except BPA male diurnal period (control females: *P* < 0.0001, BPA females: *P* < 0.05, control males nocturnal: *P* < 0.0001, control males diurnal: *P* < 0.05, BPA males nocturnal: *P* < 0.05, Wilcoxon rank sum test). The BPA-exposed female group showed significantly lower values for the average number of total visits and visits without drinking during the diurnal period (*P* < 0.01, Tukey's HSD). However, there was no difference shown between the male groups.

**Table 1:** Average number of corner visits during all of the IntelliCage sessions

	Nocturnal (1 day average)			Diurnal (1 day average)		
		
Visit	Total	WoD	WD	Total	WoD	WD
CNT-F	170.8 ± 27.7 ****	121.8 ± 24.6 ****##	49.0 ± 6.4 ****	31.2 ± 6.7	21.6 ± 4.8 ##	9.6 ± 2.8
BPA-F	152.2 ± 56.0 ***	101.7 ± 47.9 **#	50.5 ± 9.7 ***	21.6 ± 8.6 †	13.9 ± 5.7 #†	7.7 ± 3.2
CNT-M	143.3 ± 42.0 ****	91.7 ± 38.3 ****##	51.6 ± 9.2 ****	31.0 ± 15.3	17.9 ± 9.4 #	13.1 ± 7.4
BPA-M	149.0 ± 33.7 ***	95.1 ± 31.0 **#	53.8 ± 8.5 ***	30.2 ± 8.7	17.7 ± 7.9	12.5 ± 4.5

Values represent mean ± SD. CNT, control; BPA, bisphenol A; WoD, without drinking; WD, with drinking.

** *P* < 0.01,

*** *P* < 0.001,

**** *P* < 0.0001 (nocturnal vs. diurnal, Wilcoxon rank sum test).

# *P* < 0.05,

## *P* < 0.0001 (drinking vs. without drinking, Wilcoxon rank sum test).

† *P* < 0.01 (treatment, Tukey's HSD).

**Figure 1:**
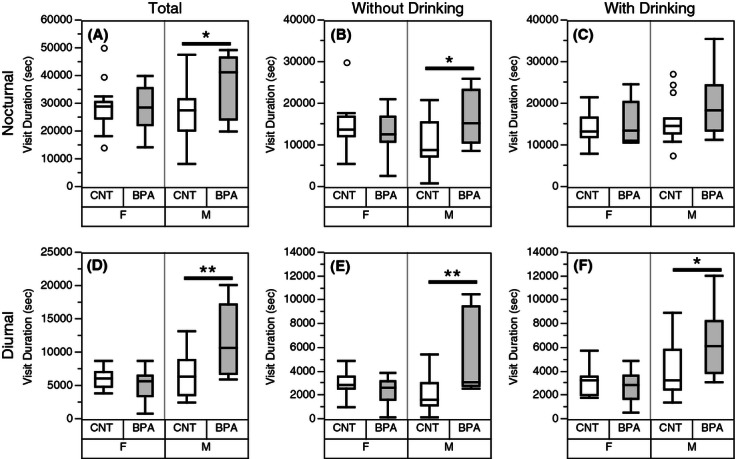
Corner visit duration was disrupted in male mice throughout the sessions: The upper rows (A, B, C) and the lower rows (D, E, F) show nocturnal (16 days total) and diurnal (15 days total) results, respectively. The left column (A, D) shows the total visits, the center column (B, E) shows without drinking visits, and the right column (C, F) shows drinking visit results. The bisphenol A (BPA)-exposed male group stayed significantly longer at the corner, compared with the control male group (A, B, D, E, F). The open circle plots represent outliers. *P < 0.05, **P < 0.01.

While no difference was shown in the number of visits by male animals between the control and the BPA groups, a significant difference was shown in the visit durations between those male groups during the nocturnal period without drinking (Fig. 1B), but not with drinking (Fig. 1C). During the diurnal period, visit duration with or without drinking was higher in the BPA males, compared with control males (Fig. 1E and F), whereas females showed no changes between the control and BPA groups. BPA-exposed males stayed at corners for a significantly longer period of time, compared with the control animals in almost all situations, whereas the female animals did not show any significant differences (Fig. 1A and D).

### Nose poke

Nose poke behavior was one of the other parameters we were able to assess with the IntelliCage. We thus analyzed nocturnal nose poke indices; the total number of nose pokes, the average number of nose poke per visit, and the ratio of the number of nose pokes with drinking to the total number of nose pokes. The results showed no significant difference between the BPA-exposed groups and the controls. In regard to sex differences, the female mice performed nose pokes more frequently than the male animals (females: 6820 ± 1669, males: 4918 ± 1218, *P* < 0.0001, Wilcoxon rank sum test). The average number of nose pokes per visit was significantly higher in the females, compared with the males (females: 2.7 ± 0.6, males: 2.2 ± 0.4, *P* < 0.01, Wilcoxon rank sum test). The ratio of the number of nose pokes with drinking to the total number of nose pokes was higher in the male animals, compared with the females (females: 17.9 ± 5.7, males: 28.3 ± 13.1, *P* < 0.0001, Wilcoxon rank sum test).

### Corner preference

The ratio of the number of visits to each corner to an animal's total number of corner visits represents an individual's preference for a specific corner. While there were no large differences between these ratios for the total visits, we noticed that many cohorts showed a bias for drinking corners.

We exploited two indices in order to compare the bias level between groups, a “Preference Bias” that showed how large an individual bias was, and an individual Preference Bias for animal(*j*) is defined as follows:


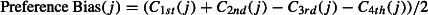


where *C*_1*st*_(*j*) is the largest corner visit ratio for animal (j). *C*_2*nd*_(*j*), *C*_3*rd*_(*j*), *C*_4*th*_(*j*) are the second, third, fourth ratio, respectively. For each case (total, with/without drinking), the animal(*j*)'s corner(*i*) visit ratio can be calculated as,


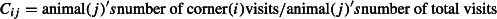


(Both visit numbers are qualified for each specific case.) The second index was a “Preference Variance” that represents how largely the bias varied within a cohort. The individual Preference Variance is defined as the Euclidean distance between the individual corner visit ratio within each case (*C*_*ij*_) and the median value of *C*_*ij*_ within the cohort (*C*_*iM*_):


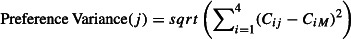


Both the Preference Bias and the Preference Variance were calculated within the cohort.

**Figure 2:**
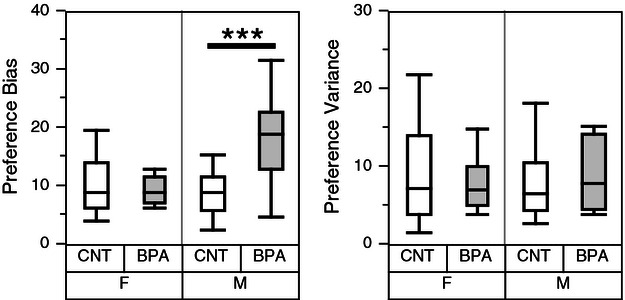
Boxplot of preference bias (left) and variance (right) in the visit with drinking: The bisphenol A (BPA)-exposed male group showed a significantly stronger bias, compared with the control group. There were no significant differences in the preference variance in either sex. ***P < 0.001.

The BPA-exposed males showed a significantly higher bias than the control males in the visit with drinking (Fig. 2). On the other hand, the Preference Variance values did not differ significantly between the male groups. It can be interpreted that a higher Preference Bias value associated with a similar Preference Variance value suggests stronger cohesiveness in terms of the corner preference. The female groups showed no significant differences for either index.

### Different-animal visit intervals

We considered the “Corner preference in the cohort” might reflect behavioral cohesiveness. In order to investigate cohort cohesiveness further we evaluated visit interval following other animal. We defined “Random Interval” for corner(*i*) as *RanINT*_*i*_ = *T*/*N*_*i*_, where *T* means total experiment time and *N*_*i*_ means total number of visits for corner(*i*) of all animals under all cases. “Different-Animal Visit Interval Rate” for corner(*i*) of animal(*j*) is calculated as follows:


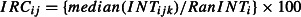


where *INT*_*ijk*_ is the interval time (the end of the previous visit – the beginning of this visit) of visit(*k*) of animal(*j*), following the preceding other animal. (Each visit is qualified to the specific case based on current visit.) Then the individual Different-Animal Visit Interval Rate can be defined as *IR*_*j*_ = *mean*(*IRC*_*ij*_). The Different-Animal Visit Interval Rate represents how fast the animal concerned visits the same corner the preceding animal visited.

**Figure 3:**
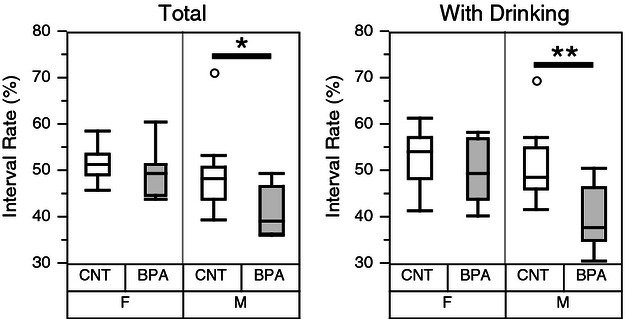
Boxplot of the nocturnal different-animal visit interval rate for the total visits (left) and the drinking visits (right): The bisphenol A (BPA)-exposed male group showed a smaller interval rate than the control group. The difference in the drinking visits was more obvious. The open circle plots represent outliers. *P < 0.05, **P < 0.01.

Figure 3 shows boxplots of the nocturnal different-animal visit interval rate. The BPA-exposed male animals showed significantly lower values than the control group in both the total visits and drinking cases. The difference between the BPA male group and the control group without drinking was not significant (data not shown). The female groups showed no significant differences in any case.

## Discussion

In this study, mice exposed to BPA during the embryonic and lactational period showed differences in several behavioral indices. BPA-exposed females visited a corner without drinking less frequently during the light cycle, compared with the control female mice. BPA-exposed males, stayed at a corner longer in almost all cases (except the nocturnal drinking case), showed a stronger preference bias and a shorter different-animal visit interval, compared with the control mice. It is worthy of mentioning that we did not find any significant differences in the maternal behavior during the pregnant and lactational periods by BPA treatment.

It has been reported that BPA exposure perturbs the neurotransmitter systems. Maternal exposure to low doses of BPA caused an increase in the levels of dopamine and its metabolite in the caudate/putamen and dorsal raphe nucleus, as well as an increase in serotonin and its metabolite in the caudate/putamen, dorsal raphe nucleus, thalamus, and substantia nigra at P3W and/or P14-15W (Nakamura et al. 2010). The density of tyrosine hydroxylase (TH)-immunoreactive neurons in the substantia nigra was significantly decreased in female mice by fetal and neonatal exposure to low-dose BPA (Tando et al. 2007). Some studies have suggested that BPA exposure perturbs reward pathways. Female mice treated with both a low and a high dose of BPA-mixed food maternally showed an enhanced morphine-induced place preference and hyperlocomotion (Narita et al. 2006), while in another study, gestational exposure to BPA diminished the d-amphetamine-induced conditioned place preference in female mice (Laviola et al. 2005).

The results of this study, showing a stronger bias for a drinking corner in BPA-exposed males, might be a consequence of disrupted reward pathways. In another study, which included an impulsivity test, rats perinatally exposed to BPA were associated with a higher marked preference for the “large and delayed (LAD)” reinforcer in both sexes and showed a delay to shift toward the “immediate and small (IAS)” reinforcer as the length of the delay was increased (Adriani et al. 2003). These results suggest that BPA-exposed animals might have perseverance toward reward and might be less prone to change their related behavior. The stronger bias for a drinking corner we observed in BPA-exposed males may be consistent with the results of that previous report with the impulsivity tests. On the other hand, we observed the reduction in the number of diurnal visits without drinking in females. It may be so that the exploratory drive is reduced or that the corners are more aversive for the BPA-treated female mice. In addition, the longer stays at corners seen in BPA-exposed males might be a consequence of perseverance to rewards.

Wolstenholme et al., reported that juvenile mice, gestationaly exposed to BPA, spent more time sitting next to each other, but less time engaging in direct interaction, compared with control mice (Wolstenholme et al. 2012). In addition, gestational exposure to BPA altered contact behavior (nose-to-nose contact and approaching) in juvenile mice. The alterations in social behavior were not sexually dimorphic but influenced by in utero BPA exposure (Wolstenholme et al. 2011, 2012). In the present study, the visit interval following preceding animals in BPA males was shorter than that of control males, which suggested that BPA-exposed males might be influenced by surrounding animals more than the control animals. Furthermore, this influence might appear more intensely in reward-related situations. The results of our Preference Bias and Preference Variance analysis suggested a similar disposition. BPA-exposed males showed a larger Preference Bias than control males and a Preference Variance comparable to control males. From a mathematical viewpoint, given a larger Preference Bias, a comparable Preference Variance means stronger cohesiveness.

The important finding of our study was that prenatal and lactational BPA exposure might affect mice motivational behavior in a social setting differently in males and females. Further studies are necessary to evaluate the underlying mechanisms of the behavioral effects of prenatal and lactation exposure to low doses of BPA.

## Conclusion

Prenatal and lactational exposure to low doses of BPA-altered mice motivational behavior in a social setting using IntelliCage, which might be related with perturbed reward pathway. Further biochemical analysis of brains from the tested mice could provide more information to substantiate our present results.
